# Haemolytic uraemic syndrome associated with non shiga toxin-producing *Escherichia coli* bacteraemia: a case report

**DOI:** 10.1186/s12882-019-1357-3

**Published:** 2019-05-07

**Authors:** Stéphane Bally, Jacques Fourcade, Véronique Frémeaux-Bacchi

**Affiliations:** 1 Service de Néphrologie Dialyse, Centre Hospitalier Métropole Savoie, Place Lucien Biset, BP 31125, 73 011 Chambery, Cedex France; 2 Service d’Immunologie Biologique, Hôpital Européen Georges Pompidou (AP-HP), 20 rue Leblanc, 75 908 Paris, Cedex 15 France

**Keywords:** Complement variant, *Escherichia coli*, Hemolytic uremic syndrome, Infection

## Abstract

**Background:**

Haemolytic uraemic syndrome (HUS) is a thrombotic microangiopathy (TMA) characterized by predominant renal involvement. Several types of HUS can be distinguished: the most frequent « typical » HUS, due to shiga toxin producing *Escherichia coli* (STEC), “atypical” HUS due to complement alternative pathway dysregulation and “secondary” HUS associated with various diseases/conditions, the classification of which is still subject to debate.

**Case presentation:**

We report a case of HUS following E.coli prostatitis and bacteraemia in an adult male. He presented with severe renal and neurological involvement. Initially considered as a “typical” HUS, the condition was treated by antibiotics. No other specific treatment for HUS was administered. The outcome was favorable. We eventually identified a non shiga toxin producing E.coli. Genetic testing of the complement alternative pathway revealed a rare – potentially pathogenic – variant of factor H. This constitutes a possible factor of susceptibility for atypical HUS, suggesting that E.coli infection may be the trigger.

**Conclusion:**

This case raises the question of complement exploration for HUS associated with infections, in order to classify such cases of HUS in accordance with their underlying pathophysiological mechanisms.

## Background

Thrombotic microangiopathies (TMA) are rare disorders characterized by an initial endothelial cell injury, and by the triad of mechanical haemolytic anaemia, thrombocytopenia and ischemic organ injury. Haemolytic uraemic syndrome (HUS) is a TMA characterized by predominant renal involvement and normal ADAMTS13 activity (> 10%), which excludes thrombotic thrombocytopenic purpura (TTP).

There are several types of HUS and their classification evolves with elucidation of their underlying pathophysiological mechanisms: 1) Infection-related HUS, mainly « typical » HUS following intestinal infection due to shiga toxin-producing *Escherichia coli* (STEC), which is the most frequent form. Other infections, such as Streptococccus pneumoniae, Influenza A, HIV, are involved more rarely. 2) Atypical HUS (aHUS), due to an acquired (auto-antibodies) or a constitutional dysregulation of complement alternative pathway, which is found in more than 60% of cases. 3) Secondary HUS, alongside coexisting diseases or conditions: drugs, malignancies, autoimmune diseases, pregnancy. 4) Other rare genetic forms of HUS are due to Cobalamin C and diacylglycerol kinase ε deficiencies. In 30% of cases the mechanism is unknown.

STEC-HUS and aHUS account for 85–90 and 5% respectively of cases of HUS in children. Their respective frequency is not well documented in adults [1, 2].

Typical HUS usually follows a STEC intestinal infection, identified via stool cultures, polymerase chain reaction (PCR) for *Stx* genes encoding for shiga toxins, or detection of anti-lipopolysaccharide (LPS) antibodies in serum.

The genetics of « atypical » complement-HUS is complex. Rare or common variants with established or highly probable functional consequences are a risk factor for developing the disease. Pathogenic variants have been identified in more than 60% of cases in one of the 8 genes encoding for C3 and factor B forming alternative C3 convertase, or for one of the 3 regulating proteins (factor H, factor I and MCP, *Membrane Cofactor Protein* or CD46) and in CFHR5, DGKe or the gene of thrombomodulin. Common variants in FH and MCP genes increase the risk of developing the disease by 2 to 5 times. In most cases, a trigger is necessary to initiate the disease.

We report a case of HUS in an adult male, following non STEC E.coli prostatitis and bacteraemia. Genetic testing of the complement alternative pathway revealed a rare variant of factor H.

## Case presentation

A 58-year-old man was hospitalized for haemorrhoid surgery. His medical history featured only hypercholesterolaemia.

On day 1 after surgery, he developed fever and symptoms of prostatitis, with no digestive symptoms. Following blood and urine cultures, antibiotherapy (ofloxacin and gentamycin) was initiated. Platelet count was 100 G/L (normal before surgery), haemoglobin (Hb) was normal (14.5 g/dL). Renal function was normal (serum creatinine = 1.02 mg/dL).

Urine and blood cultures came back positive for *Escherichia coli* and ofloxacin was continued. No E.coli was found in the stools (culture and PCR).

On day 4, platelet count decreased to 27 G/L, without anaemia, and creatinine rose to 1.75 mg/dL, but the patient had urinary retention.

Day 6, although the infection was under control and the patient had remained haemodynamically stable throughout (blood pressure 120/62 mmHg), he developed acute kidney injury (AKI) with anuria (creatinine = 7.36 mg/dL) and neurological signs which included confusion, hallucinations, anterograde amnesia, static cerebellar syndrome and transient motor deficit of the left lower limb. The renal CT-scan was normal, as was cerebral magnetic resonance imaging (MRI). Laboratory tests showed: Hb = 11.8 g/dL, haptoglobin = 1.53 g/L, LDH = 2615 U/L (upper limit 480 U/L), platelet count = 61 G/L.

This renal and neurological presentation was initially attributed to sepsis and possible drug toxicity (antibiotics). Haemodialysis was started and antibiotherapy modified to ceftriaxone.

On days 9–12, the patient’s neurological state worsened: he presented seizures, controlled using anti epileptic treatment. The spinal tap was normal. At this point Hb had dropped to 8 g/dL, LDH remained elevated (1265 U/L) and schistocytes 3% were detected. Platelet count, however, normalized (250 G/L). ADAMTS13 activity was normal (38%, with FRETS-VWF73 technique), thus excluding a diagnosis of TTP. Antinuclear antibodies and ANCA were negative.

A hypothesis of HUS associated with “non-intestinal” STEC infection was made.

An initial complement work-up revealed no abnormalities (Table 1).

**Table 1:** Complement testing

Protein and technique	Result	Normal range
CH50 (hemolytic assay)	136%	70–130
C3 antigen (nephelometry)	1430 mg/L	660–1250
C4 antigen (nephelometry)	283 mg/L	93–380
Factor B antigen (nephelometry)	195 mg/L	90–320
Factor H antigen (ELISA)	149%	65–140
Anti-factor H antibodies (isotype IgG) (ELISA)	negative	
Factor I antigen (ELISA)	137%	70–130
Membrane expression of CD46 (MCP) at the surface of granulocytes CD33+ (Beckman Coulter cytometer, type Navios)	18.8	13.0–19.0

**Fig. 1:**
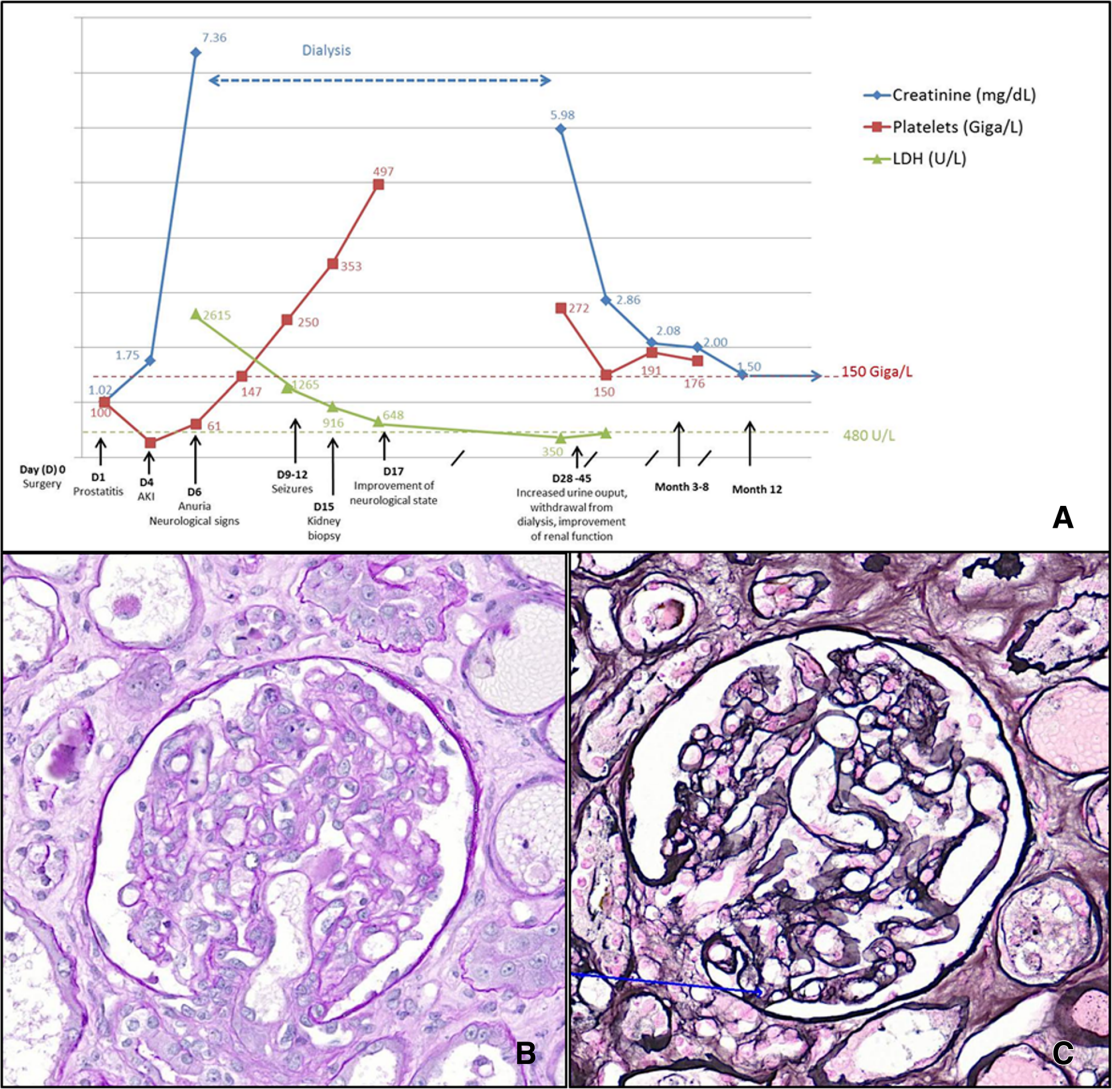
Clinical and biological course, and anatomopathological features. **a** shows the clinical and biological course. Light microscopy study of the kidney biopsy specimen shows TMA features: (**b**) endothelial cells swelling with periodic acid-Schiff stain (× 20) and (**c**) double contours of the glomerular basement membrane (blue arrow) with silver stain (× 20). Fibrin and platelet thrombi were also observed in glomerular capillaries

A kidney biopsy, performed on day 15, confirmed the diagnosis of HUS. Light microscopy showed 13 permeable glomeruli with endothelial cell swelling, widening of the subendothelial space, and double contours of the glomerular basement membrane. Fibrin and platelet thrombi were observed in glomerular capillaries. Images of regenerating tubular necrosis were also observed. An immunofluorescence study revealed no significant immune deposits (Fig. 1).

Haemolysis subsequently disappeared and the patient’s neurological state improved. Although urine output increased, the patient remained on dialysis.

While waiting to identify the type of *E. coli* involved, we were unable to exclude a diagnosis of non-intestinal STEC-HUS. In view of the favorable outcome, the patient did not receive any plasmatherapy, or complement inhibitor. However, we were able to discontinue dialysis after 1 month as renal function improved.

When testing for anti-LPS antibodies in serum was repeated 3 weeks after disease onset, no E.coli typically associated with HUS in France was found (detection of IgM and IgA (‘line-blot’) anti-LPS antibodies in the serum, for serogroups O26, O55, O91, O103, O111, O128, O145, O157). Moreover, screening for virulence factors of the E.coli found in blood cultures came back negative (*stx1* and *stx2* gene amplification negative; *eae* and *ehxA* gene amplification negative; *fyuA, hlyC, sfa/foc, papC, iucC, papGIII, cnf1, papGII, iroN, vat, clbB* genes negative).

After 3 months, serum creatinine was 2 mg/dL and proteinuria was 0.4 g/day. After 1 year, serum creatinine remained stable at 1.5 mg/dl, with normal blood pressure and clinical state. No recurrence has been observed over a 3 year period.

Screening for rare variants in complement alternative pathway genes was performed (Technique: EUrenOmics NGS panel) and showed the presence of a heterozygous variant in the gene encoding complement factor H (p.Val215Ile; c.643 G > A). This variant is located in the N-terminal domain of the protein (SCR4) and is not present in the ExAc data base (http://exac.broadinstitute.org/).

## Discussion and conclusions

We describe a case of HUS associated with an E.coli bacteraemia with a urinary starting point, without diarrhea and without the presence of E.coli in the stools. This clinical presentation is rare, although cases of « typical » STEC-HUS without diarrhea – in particular cases associated with acute pyelonephritis and sometimes bacteraemia – have been reported [3–5]. However, in this case, E.coli was not a STEC. Testing for anti-LPS antibodies did not identify any E.coli typically associated with HUS in France. That was not enough to exclude certain emerging serotypes such as O104:H4 or O80:H2, which can be responsible for similar clinical presentations [6]. But further testing for shigatoxines Stx1 and Stx2 (PCR) and screening for any other virulence factor of the E.coli found in the blood cultures were negative.

A transient activation of complement is observed in patients with STEC-HUS [7, 8]. Some of them carry genetic factors of susceptibility for aHUS, supporting the hypothesis of a multiple-hit model, with infection playing the role of trigger, on the grounds of genetic predisposition [9, 10].

In our case, we hypothesize that the non shiga toxin-producing E.coli sepsis was the trigger for HUS.

An extensive biochemical complement analysis proved normal (Table 1). This type of finding has been reported in number of aHUS cohorts. In the French Cohort, C3 level is lower in only 40% of cases. Almost half of pathogenic variants have functional consequences, which have no impact on antigenic levels. So this does not exclude the diagnosis [11, 12].

We finally carried out screening of complement alternative pathway genes, and this revealed a heterozygous variant in the gene encoding complement factor H (p.Val215Ile; c.643 G > A). Factor H (FH) is the key regulator of the alternative pathway (AP). The CFH variant located in SCR4 of the protein is not present in the large cohort of healthy donors. This suggests a frequency of under 0.001%. Its pathogenicity has not been reported. The SCR1–4 region is the only C3b binding site which acts as a cofactor for FI to cleave C3b and as an accelerant of AP C3-convertase decay. In this respect, the CFH variant p.Val215Ile may impair complement regulatory activity on surfaces. However the functional implications of the CFH variant warrant further investigation but could explain this serious presentation of non STEC-HUS.

Clinical outcome was finally favorable without complement blockade, with a residual mild chronic kidney disease. However the presence of a complement gene variant prompts us to maintain regular monitoring, particularly during periods of infection, in order to detect any recurrence of HUS rapidly.

This case raises the question of complement testing in patients with infection related-HUS. There is little data in the literature regarding the existence of genetic factors of susceptibility in cases such as these. Interactions between infection and HUS are dual. On the one hand, infection on its own can induce endothelial injury and complement activation, leading to TMA. On the other hand, infection – like other conditions such as pregnancy – can be a trigger for aHUS in susceptible patients [11–13]. So, it has been demonstrated that cases of pregnancy related-HUS share the same genetic factors of predisposition as so-called ‘primary’ aHUS; pregnancy is therefore considered to be a trigger for aHUS [14].

This issue is important because in the first case HUS will normally not recur, whereas in cases of genetic predisposition, HUS may do so.

Further studies assessing the implications of complement dysregulation in infection-related HUS are, consequently, warranted.

## Abbreviations

aHUS: Atypical haemolytic uraemic syndrome
AKI: Acute kidney injury
AP: Alternative pathway
CFH: Complement factor H
CFI: Complement factor I
G: Giga
Hb: Haemoglobin
HUS: Haemolytic uraemic syndrome
L: Liter
LPS: Lipopolysaccharide
MRI: Magnetic resonance imaging
PCR: Polymerase chain reaction
STEC: Shiga toxin-producing *Escherichia coli*
TMA: Thrombotic microangiopathy
TTP: Thrombotic thrombocytopenic purpura
